# Exercise Training Characteristics and Psycho-Bio-Chemical Outcomes in Individuals with Addictive Behaviours: A Scoping Review

**DOI:** 10.3390/jfmk11030311

**Published:** 2026-08-10

**Authors:** Guilherme Eustáquio Furtado, Maria Inês Marques, António Rodrigues Sampaio, Daniel Duarte, Susana Ramos, Eduardo Carballera, Marta Sevilla-Sanchez, Edson Eduardo, Sónia Mateus, Patrícia Coelho, Francisco Rodrigues

**Affiliations:** 1 Applied Research Institute, Polytechnic Institute of Coimbra, Rua da Misericórdia, Lagar dos Cortiços–S. Martinho do Bispo, 3045-093 Coimbra, Portugal; marta.sevilla@udc.es; 2 SPRINT—Sport Physical Activity and Health Research & INnovation Center, Polytechnic University of Coimbra, Rua Dom Joao III—Solum, 3030-329 Coimbra, Portugal; 3 Human Performance and Exercise for Health Lab (HuPExH Lab), Universidad de La Laguna, 38200 San Cristóbal de La Laguna, Spain; ecarball@ull.edu.es; 4 Dr. Lopes Dias School of Health, Polytechnic University of Castelo Branco, 6000-084 Castelo Branco, Portugal; ines12003@gmail.com (M.I.M.); patriciacoelho@ipcb.pt (P.C.); 5 Research Center in Sports Sciences, Health Sciences and Human Development (CIDESD), University of Maia (UMaia), Avenida Carlos de Oliveira Campos, Castêlo da Maia, 4475-690 Maia, Portugal; arsampaio@umaia.pt (A.R.S.); dfduarte@ipmaia.pt (D.D.); 6 Research Center of the Polytechnic Institute of Maia (N2i), Maia Polytechnic Institute (IPMAIA), 4475-690 Maia, Portugal; 7 Faculty of Sport Sciences and Physical Education, University of Coimbra, Pavilhão 3, 3040-248 Coimbra, Portugal; susanaramos@fcdef.uc.pt (S.R.); edsongaspar76@gmail.com (E.E.); 8 Department of Physical Education and Sport, Faculty of Sports Sciences and Physical Education, University of A Coruna, 15071 A Coruna, Spain; 9 Research Group of Physical Activity and Health, Faculty of Physical Education and Sport, Pedagogical University of Maputo, Av. Eduardo Mondlane No. 955, Maputo CP 2107, Mozambique; 10 Sport Physical Activity and Health Research & Innovation Center (Sprint), Polytechnic University of Castelo Branco, 6000-084 Castelo Branco, Portugal

**Keywords:** exercise training, addictive behaviours, substance use disorder, behavioural addiction, FITT principles, craving, neurobiology, scoping review

## Abstract

Addictive behaviours are associated with substantial neurobiological, psychological, and behavioural impairments. Exercise training has emerged as a potential complementary strategy in addiction treatment; however, the characteristics of exercise interventions and their associated outcomes remain insufficiently characterized. This scoping review (ScR) was conducted according to the Joanna Briggs Institute methodology and reported in accordance with the PRISMA-ScR guidelines. Searches were conducted across three electronic databases for studies published between January 2015 and March 2025. Eligible studies investigated structured exercise training interventions in individuals with substance-related or behavioural addictions. Data were extracted regarding study characteristics, addiction type, exercise modality, FITT parameters, and neurobiological, biochemical, psychological, behavioural, and addiction-related outcomes. Five human intervention studies met the inclusion criteria, all addressing substance-related addictions, including alcohol use disorder, methamphetamine use disorder, and polysubstance dependence. Three studies evaluated acute exercise interventions, whereas two investigated structured exercise programmes lasting 8–12 weeks. Aerobic exercise was the most frequently investigated modality, although combined aerobic and resistance training, yoga, cycling, soccer-based exercise, circuit training, and functional exercise were also identified. Exercise intensity ranged from moderate to vigorous, with substantial variability in frequency, session duration, intervention period, and reporting of progression, supervision, and adherence. Reported outcomes included dopamine-related measures, mood, anxiety, depressive symptoms, self-esteem, affect, and craving. The available evidence suggests potentially relevant effects of exercise across neurobiological, psychological, and addiction-related domains, but the limited number and heterogeneity of studies preclude conclusions regarding the effectiveness of specific exercise prescriptions. The current evidence base on structured exercise training in addictive behaviours remains limited and heterogeneous and is restricted, among the eligible intervention studies identified, to substance-related addictions. Exercise may represent a promising complementary approach, but evidence is insufficient to establish optimal exercise modalities, FITT prescriptions, or sustained clinical benefits. Future research should prioritize adequately powered longitudinal randomized controlled trials, standardized reporting of exercise prescription and adherence, and comprehensive assessment of clinically relevant addiction-related outcomes.

## 1. Introduction

Addictive behaviours represent a major public health concern due to their profound effects on physical health, mental well-being, social functioning, and quality of life [1]. Traditionally associated with substance-related disorders such as alcohol, nicotine, and illicit drug dependence, the concept of addiction has progressively expanded to include behavioural addictions, including problematic smartphone use, internet gaming disorder, compulsive buying, and gambling disorder [2].

These conditions share common neurobiological and psychological mechanisms, particularly involving reward processing, impulse control, emotional dysregulation, and maladaptive reinforcement pathways [3]. Consequently, addictive behaviours are increasingly recognized as multidimensional disorders requiring comprehensive and integrative treatment approaches [4].

Globally, substance-related addictions continue to contribute substantially to morbidity and mortality [5]. Alcohol consumption remains one of the leading preventable risk factors for disease burden worldwide, while psychoactive substance use continues to represent an important health concern, particularly among adolescents and young adults [6]. In parallel, the rapid digitalization of daily life has intensified the prevalence of behavioural addictions, especially among younger populations [7]. Excessive smartphone use, problematic gaming, and compulsive online behaviours have been associated with anxiety, depression, sleep disturbances, reduced physical activity, and impaired psychosocial functioning [7]. These concerns should also be considered within the broader context of emerging lifestyle-related health risks during childhood and adolescence, as early alterations in cardiovascular risk profiles and excess body weight have been documented in school-aged populations [8,9]. These trends highlight the need for preventive and therapeutic strategies capable of addressing both substance-related and behavioural forms of addiction.

The pathophysiology of addictive behaviours involves complex interactions between neurobiological, psychological, and environmental factors. Dysregulation of dopaminergic pathways, particularly within the mesocorticolimbic reward system, plays a central role in the development and maintenance of addictive behaviours [10]. Alterations in neurotransmitters and stress-related biomarkers, including dopamine, serotonin, cortisol, and brain-derived neurotrophic factor (BDNF), have been associated with craving, compulsive behaviours, mood disturbances, and relapse susceptibility [11]. These biomarkers are increasingly investigated as potential indicators of treatment response and neurobiological adaptation [12].

Exercise training has emerged as a promising non-pharmacological approach that may complement established strategies for the management of addictive behaviours [13,14]. Exercise-induced adaptations may influence several mechanisms implicated in addiction, including reward sensitivity, stress regulation, emotional processing, and neuroplasticity [13,15]. Available evidence suggests that exercise interventions may be associated with changes in craving, anxiety, depressive symptoms, self-esteem, mood, cognitive function, and overall well-being in individuals with addictive behaviours [13,16,17,18,19]. However, the characteristics of the exercise interventions and the outcomes assessed vary substantially across studies.

Exercise training may also exert systemic physiological effects that are relevant to individuals with addictive behaviours, including improvements in physical fitness and cardiometabolic health [20,21,22]. Nevertheless, the specific contribution of exercise prescription characteristics to the observed outcomes in populations with addictive behaviours remains insufficiently characterized. In particular, studies differ considerably in exercise modality, frequency, intensity, duration, supervision, and overall intervention length, limiting direct comparison between interventions and the translation of findings into clinical practice.

Furthermore, while previous reviews have investigated the general effects of physical activity or exercise on substance use disorders [20,21], limited evidence synthesis has comprehensively mapped structured exercise training interventions across both substance-related and behavioural addictions while simultaneously characterizing exercise prescription according to the FITT principles (frequency, intensity, time, and type) and documenting the biochemical, psychological, behavioural, and clinical outcomes assessed.

The restriction of the present review to studies published from 2015 onward was adopted to capture contemporary exercise interventions within a period characterized by the increasing recognition of behavioural addictions, rapid expansion of research on problematic digital behaviours, and continued development of exercise-based approaches to addiction management. Nevertheless, this temporal restriction may have excluded earlier intervention studies and should therefore be considered when interpreting the scope of the available evidence.

Importantly, the diversity of study populations, addictive behaviours, exercise modalities, intervention protocols, and outcome measures makes a scoping review particularly appropriate. Rather than primarily evaluating intervention effectiveness, a scoping review allows the available evidence to be mapped systematically, intervention characteristics to be described, and knowledge gaps to be identified. This approach is particularly relevant to the present topic because the extent and nature of exercise prescription across different addictive behaviours remain insufficiently characterized.

Therefore, this scoping review aimed to map structured exercise training interventions in individuals with substance-related and behavioural addictions, with particular emphasis on exercise prescription according to the FITT principles and the neurobiological, psychological, behavioural, and clinical outcomes assessed. By characterizing how exercise interventions have been designed and implemented and identifying the outcomes investigated, this review seeks to describe the current evidence base and highlight gaps that may inform future research and intervention development.

## 2. Materials and Methods

### 2.1. Review Design

This scoping review was conducted in accordance with the methodological framework proposed by the Joanna Briggs Institute (JBI) Manual for Evidence Synthesis for Scoping Reviews [16] and reported following the Preferred Reporting Items for Systematic Reviews and Meta-Analyses Extension for Scoping Reviews (PRISMA-ScR) guidelines [23].

A scoping review methodology was considered appropriate because the available evidence regarding structured exercise training interventions in individuals with addictive behaviours is heterogeneous in terms of populations, addiction types, exercise modalities, intervention characteristics, and outcomes assessed. The primary purpose of this review was therefore to map and characterize the existing evidence on structured exercise training interventions in individuals with substance-related and behavioural addictions, with particular emphasis on exercise prescription according to the FITT principles and the outcomes assessed.

No review protocol was prospectively registered. Although prospective protocol registration is not mandatory for scoping reviews, the absence of a registered protocol may represent a limitation because it reduces the availability of an a priori documented methodological record against which the review process can be compared.

### 2.2. Review Question

The review question was developed according to the Population–Concept–Context (PCC) framework recommended by the JBI methodology for scoping reviews [24]:

- Population (P): Individuals with substance-related or behavioural addictions.
- Concept (C): Structured exercise training interventions, including their prescription according to the FITT principles, and the physiological, biochemical, psychological, behavioural, and clinical outcomes assessed.
- Context (C): Any clinical, institutional, community, academic, rehabilitation, or other setting in which structured exercise interventions were implemented.

Accordingly, the primary review question was as follows:

What structured exercise training interventions have been implemented in individuals with substance-related or behavioural addictions, how have these interventions been characterized according to the FITT principles, and which outcomes have been assessed?

Secondary questions addressed the following: (i) the types of addictive behaviours investigated; (ii) the exercise modalities and prescription characteristics reported; (iii) the biochemical, physiological, psychological, behavioural, and clinical outcomes assessed; and (iv) the main evidence gaps requiring further investigation.

### 2.3. Eligibility Criteria

Eligibility criteria were established a priori according to the PCC framework and the objectives of the review. Following the critical reassessment of the eligibility criteria during the revision process, the review was restricted to human intervention studies to ensure consistency between the review question, the characterization of exercise training, and the interpretation of the findings.

#### 2.3.1. Inclusion Criteria

Studies were considered eligible if they met all of the following criteria:

(i) Included human participants with substance-related addictions (e.g., alcohol, nicotine, cannabis, cocaine, methamphetamine, opioids, or polysubstance use disorders) and/or behavioural addictions (e.g., internet gaming disorder, gambling disorder, problematic smartphone use, compulsive buying, or problematic internet use);
(ii) Investigated a structured exercise training intervention, including but not limited to aerobic training, resistance training, high-intensity interval training (HIIT), combined training, yoga, Tai Chi, or multicomponent exercise programmes;
(iii) Reported at least one characteristic related to exercise prescription according to the FITT principles (frequency, intensity, time, and/or type);
(iv) Assessed at least one physiological, biochemical, psychological, behavioural, or clinical outcome relevant to the study population or addictive behaviour;
(v) Included participants of any age, sex, or clinical status;
(vi) Employed an interventional study design, including randomized controlled trials, non-randomized intervention studies, pilot studies, feasibility studies, and other prospective exercise intervention designs;
(vii) Were published as full-text articles in peer-reviewed journals in English, Portuguese, or Spanish; and
(viii) Were published between January 2015 and March 2025

The 2015–March 2025 publication window was selected to capture contemporary evidence on exercise prescription and addiction research, including the increasing investigation of behavioural addictions and problematic digital behaviours, as well as more recent developments in exercise-based approaches and the assessment of neurobiological and psychosocial outcomes. Nevertheless, this restriction may have excluded relevant earlier intervention studies and was therefore considered when interpreting the scope of the mapped evidence.

#### 2.3.2. Exclusion Criteria

Studies were excluded if they

(i) Did not involve human participants with a substance-related or behavioural addiction;
(ii) Did not evaluate a structured exercise training intervention;
(iii) Were observational or cross-sectional studies based exclusively on self-reported or habitual physical activity without a structured exercise intervention;
(iv) Were pre-clinical or animal studies;
(v) Were study protocols without intervention results;
(vi) Did not report exercise prescription characteristics or outcomes relevant to the review objectives;
(vii) Were conference abstracts, editorials, letters, commentaries, dissertations, case reports, or review articles;
(viii) Included fewer than 10 participants;
(ix) Were not available in full text; or
(x) Were not published in English, Portuguese, or Spanish.

### 2.4. Search Strategy

A comprehensive literature search was conducted in three electronic databases: PubMed/MEDLINE, Scopus, Web of Science Core Collection. These databases were selected to provide coverage of biomedical, health, psychological, psychiatric, addiction, behavioural, and exercise science literature.

The search strategy combined controlled vocabulary and free-text terms related to three main concepts: exercise training, addictive behaviours, and relevant outcomes. Boolean operators (“AND” and “OR”), truncation, phrase searching, and database-specific indexing terms were applied according to the requirements of each database.

Reference lists of included studies and relevant reviews were also screened manually to identify potentially eligible studies not retrieved through the electronic database searches.

### 2.5. Study Selection

All identified records were exported to Zotero for reference management and duplicate removal. The study selection process was subsequently conducted in two stages by two independent reviewers: (i) screening of titles and abstracts according to the eligibility criteria; and (ii) full-text assessment of potentially relevant studies.

Disagreements between reviewers were resolved through discussion and consensus. When consensus could not be reached, a third reviewer was consulted.

The study selection process was documented using a PRISMA-ScR flow diagram [23]. Where automated tools were used to assist with the initial identification or exclusion of records, their use and purpose are described in the corresponding PRISMA-ScR flow diagram and accompanying text.

### 2.6. Data Extraction and Charting

Data extraction was performed using a standardized charting form developed specifically for this review in Microsoft Excel. Two reviewers independently checked the extracted information, and discrepancies were resolved through discussion and consensus.

The following information was extracted from each included study:

Study characteristics: authors, year of publication, country, and study design.

Participant characteristics: sample size, age, sex, type of addictive behaviour, and relevant clinical characteristics.

Exercise intervention characteristics: exercise modality/type, frequency, intensity, session duration/time, total intervention duration, and other FITT-related characteristics.

Implementation characteristics: supervision, adherence, follow-up assessments, and other relevant exercise-related variables, when reported.

Outcomes: biochemical, physiological, psychological, behavioural, and clinical outcomes assessed, together with the direction of the reported findings when available.

Participant and intervention characteristics were standardized across studies wherever sufficient information was available. When a variable was not reported by the original study, it was recorded as “not reported” rather than inferred.

### 2.7. Outcomes Assessed

Outcomes were categorized into two broad domains.

Biochemical and physiological outcomes included dopamine, serotonin, cortisol, BDNF, inflammatory markers, neurophysiological variables, physical or physiological fitness indicators, and other biological measures reported by the included studies.

Psychological, behavioural, and clinical outcomes included anxiety, depression, stress, craving, mood, self-esteem, well-being, addictive behaviour severity, relapse-related indicators, and other clinically relevant outcomes.

The categorization was descriptive and was used to facilitate evidence mapping rather than to estimate intervention effectiveness.

### 2.8. Data Synthesis and Presentation

The extracted data were synthesized using a descriptive and narrative approach consistent with the objectives of a scoping review. Quantitative information was summarized using frequencies and percentages whenever appropriate, while findings that could not be meaningfully quantified were synthesized narratively.

Studies were grouped according to the type of addictive behaviour, exercise modality, intervention characteristics, and outcomes assessed. Exercise interventions were mapped according to the FITT principles (frequency, intensity, time, and type), with additional attention to supervision, adherence, and intervention duration when reported.

For each included study, the direction of reported outcome changes was summarized descriptively using standardized indicators where appropriate. These indicators were intended to facilitate interpretation of the mapped evidence and did not represent a quantitative estimate of treatment effect.

Results were presented through consolidated summary tables and thematic narrative descriptions to identify patterns, methodological and intervention heterogeneity, research trends, and knowledge gaps.

No formal critical appraisal or risk-of-bias assessment was performed, as the primary purpose of this scoping review was to map the breadth and characteristics of the available evidence rather than to estimate intervention effectiveness.

## 3. Results

### 3.1. Search Results and Study Selection

**Figure 1:**
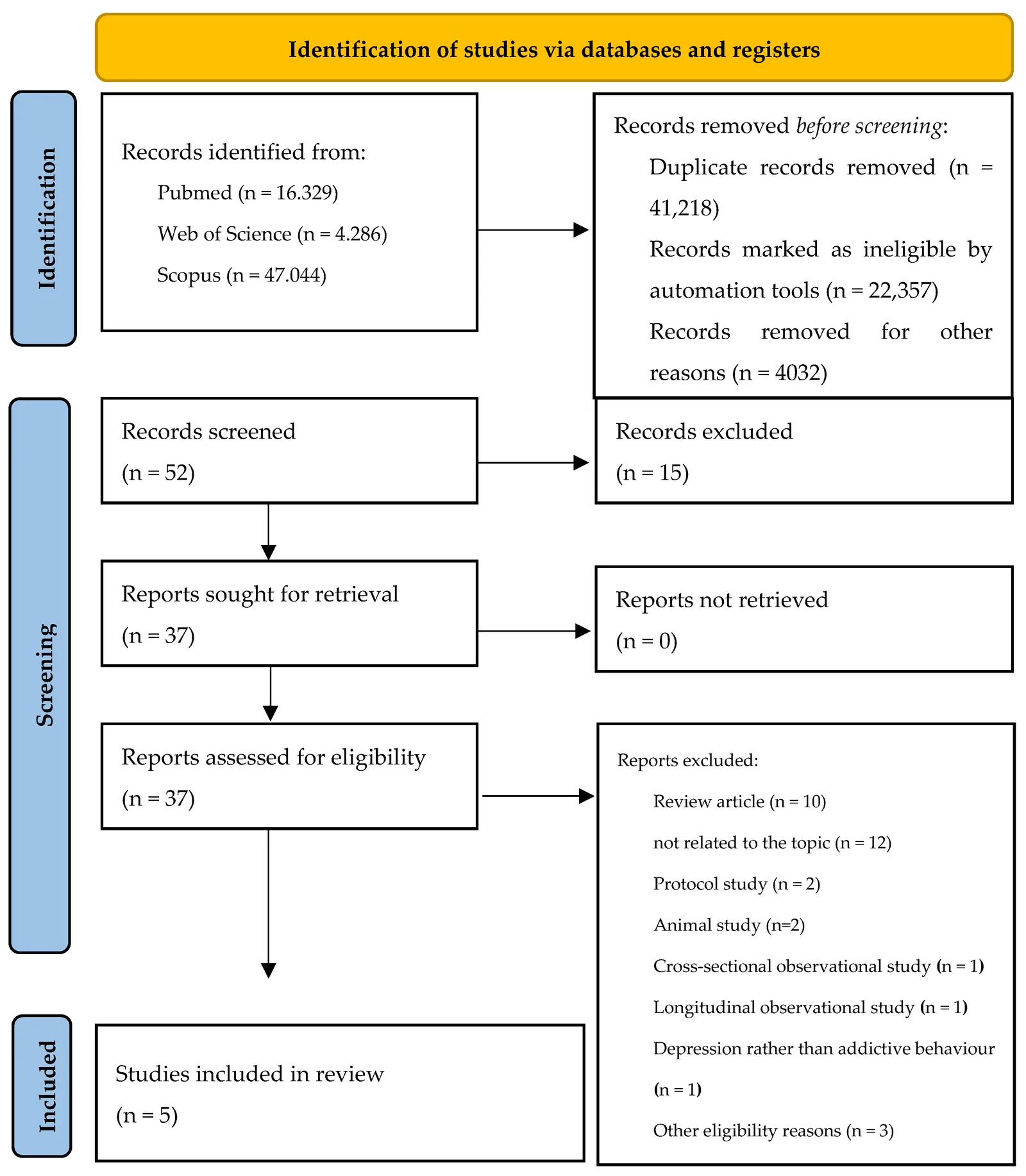
PRISMA-ScR flowchart illustrating all phases of studies included.

The study selection process was conducted in accordance with the PRISMA-ScR guidelines and is illustrated in Figure 1. A total of 67,659 records were identified through searches of the three electronic databases. Before screening, 41,218 duplicate records were removed, 22,357 records were marked as ineligible by automation tools, and 4032 records were removed for other reasons, leaving 52 records for title and abstract screening. Of these, 15 records were excluded, and 37 reports were subsequently sought for retrieval and assessed for eligibility.

Subsequently, 37 full-text articles were assessed for eligibility. Following full-text assessment, studies were excluded because they did not meet the revised eligibility criteria, including studies involving animal models, study protocols without intervention results, observational or cross-sectional designs without structured exercise interventions, and studies involving populations without an eligible addictive behaviour. In particular, one study focused on clinical depression rather than an addictive disorder and was therefore excluded.

Following this eligibility assessment, five studies met all inclusion criteria and were included in the final evidence map [25,26,27,28,29]. The updated selection process and reasons for exclusion are presented in Figure 1.

### 3.2. Characteristics of Included Studies

The five included studies investigated a range of exercise approaches in individuals with substance use disorders (SUDs), with substantial variability in exercise modality, frequency, intensity, session duration, and intervention period (Table 1). The evidence comprised three acute exercise studies [25,26,27] and two structured exercise interventions lasting several weeks [28,29].

Aerobic exercise was the most consistently represented modality. Malagodi et al. [25] examined moderate-intensity aerobic and functional exercise in hospitalized adults with SUD, whereas Hallgren et al. [27] investigated an acute bout of cycle ergometer exercise in adults with alcohol use disorder (AUD). Robertson et al. [29] evaluated a structured programme combining aerobic and resistance exercise three times per week for eight weeks in individuals with methamphetamine use disorder undergoing behavioural treatment. Welford et al. [28] investigated supervised exercise interventions, including aerobic exercise and yoga, over a 12-week period in adults with AUD. Ellingsen et al. [26] examined acute soccer-based and circuit exercise in inpatients with polysubstance dependence.

Exercise intensity varied from moderate to vigorous levels across studies. Moderate-intensity exercise was investigated in several studies [25,27,28,29], whereas Ellingsen et al. [26] examined moderate-to-vigorous exercise. The reporting of intensity was not fully standardized, with studies using heart-rate-based prescriptions, perceived exertion, or individualized descriptions. Consequently, direct comparison of exercise intensity across interventions was limited.

The duration and frequency of exercise also varied substantially. Acute interventions involved single exercise sessions ranging from approximately 12 to 45 min [25,26,27], while structured interventions extended for eight or 12 weeks [28,29]. The longer interventions generally included supervised sessions performed at least three times per week [28,29]. Information regarding adherence was inconsistently reported across studies, limiting the characterization of actual exercise exposure.

Overall, the mapped evidence demonstrates considerable heterogeneity in FITT characteristics. Although aerobic exercise was the most frequently investigated modality, no consistent exercise prescription could be identified across the included studies. This variability highlights the need for standardized reporting of exercise frequency, intensity, duration, modality, supervision, progression, and adherence in future research.

### 3.3. Exercise Training Characteristics

The included studies assessed a heterogeneous range of outcomes spanning psychological, affective, addiction-related, and neurobiological domains (Table 2). The distribution of outcomes differed according to study design and intervention duration, with acute exercise studies primarily focusing on immediate psychological and affective responses and longer interventions providing information on broader clinical or neurobiological outcomes.

Psychological outcomes were frequently investigated. Malagodi et al. [25] examined affective responses and mood state following moderate-intensity exercise in individuals with SUD. Ellingsen et al. [26] assessed affect, anxiety, and self-esteem following acute exercise in polysubstance-dependent inpatients. Hallgren et al. [27] evaluated mood and anxiety responses following an acute exercise session in adults with AUD. Welford et al. [28] assessed depressive and anxiety symptoms in the context of a 12-week exercise intervention among adults with AUD.

Addiction-related outcomes were also investigated, although less consistently. Hallgren et al. [27] specifically examined alcohol craving following acute exercise, whereas the studies by Welford et al. [28] and Robertson et al. [29] evaluated exercise within treatment contexts involving AUD and methamphetamine use disorder, respectively. The outcomes therefore varied from immediate craving responses to broader clinical and treatment-related variables.

Neurobiological assessment was most explicitly represented in the study by Robertson et al. [29], which examined striatal dopamine D2/D3 receptor availability using positron emission tomography in individuals with methamphetamine use disorder undergoing behavioural treatment. This study provides a neurobiological perspective on exercise-related adaptations within the reward system. However, the limited number of studies incorporating direct neurobiological or biochemical measurements prevents a comprehensive mapping of these mechanisms across substance use disorders.

Taken together, the five studies mapped evidence across multiple outcome domains, including affect, mood, anxiety, depressive symptoms, self-esteem, craving, and dopaminergic markers. However, the diversity of outcome measures and assessment approaches limits direct comparison between studies. The available evidence should therefore be interpreted as a map of the domains investigated rather than as evidence of consistent effects across outcomes.

### 3.4. Evidence Gaps and Heterogeneity

Several important evidence gaps were identified across the five included studies. First, all eligible studies focused on substance use disorders, particularly alcohol use disorder, polysubstance dependence, and methamphetamine use disorder. Although the original search strategy encompassed both substance-related and behavioural addictions, no eligible intervention studies addressing behavioural addictions remained after application of the predefined eligibility criteria. This indicates an important gap in the current intervention literature and limits the generalizability of the findings beyond substance-related disorders.

Second, the evidence was characterized by substantial heterogeneity in study design and exercise exposure. Three studies investigated acute exercise responses [25,26,27], whereas only two evaluated structured exercise programmes over several weeks [28,29]. This distinction is relevant because acute exercise studies primarily provide information about immediate responses, while longer interventions are more informative regarding adaptations associated with repeated exercise exposure.

Third, reporting of FITT parameters was inconsistent. Although exercise modality and session duration were generally identifiable, frequency, intensity, progression, supervision, and adherence were not uniformly reported or operationalized. This heterogeneity prevents the identification of a specific exercise prescription that could be consistently applied across substance use disorders.

The included studies also differed considerably in sample characteristics, clinical setting, addiction profile, and outcome assessment. Samples ranged from small clinical groups to larger AUD cohorts, and studies included both treatment-seeking and non-treatment-seeking populations. Such differences further limit comparability and may contribute to variability in the observed outcomes.

Finally, the evidence base remains limited in terms of sample size, intervention duration, and follow-up. Few studies incorporated direct neurobiological or biochemical assessments, and the available measures varied substantially between studies. Consequently, the present scoping review identifies a fragmented but emerging body of research investigating exercise across psychological, behavioural, addiction-related, and neurobiological domains. These findings primarily highlight where evidence currently exists and where important gaps remain, rather than establishing the effectiveness of specific exercise interventions for substance use disorders.

## 4. Discussion

The present scoping review provides a structured map of the available evidence on exercise training interventions in the context of substance-related and behavioural addictions, although all eligible intervention studies identified in this review concerned substance-related addictions, with particular emphasis on exercise prescription according to the FITT principles and the outcomes assessed. Following application of the eligibility criteria, only five human intervention studies were identified, all of which addressed substance-related addictions. This finding is relevant because it demonstrates that, despite growing interest in exercise as a complementary approach in addiction care, the intervention literature remains limited and unevenly distributed across addiction types. The evidence was also characterized by substantial heterogeneity in exercise modalities, intervention duration, intensity, participant characteristics, clinical settings, and outcome assessment. Accordingly, the principal contribution of this review is not to establish the effectiveness of exercise, but to clarify how exercise interventions have been investigated to date and to identify important gaps in their characterization and evaluation.

### 4.1. Exercise Prescription and FITT Characteristics

The included studies demonstrated considerable variability in exercise prescription. Aerobic exercise was frequently investigated, although combined aerobic and resistance training, yoga, cycling, soccer-based exercise, circuit training, and functional exercise were also represented. Exercise intensity ranged from moderate to vigorous, while intervention frequency varied from single acute sessions to repeated supervised sessions over several weeks. Intervention duration similarly varied substantially, with some studies evaluating acute responses and others implementing structured programmes over several weeks.

This heterogeneity is important because exercise prescription characteristics were not consistently reported across studies. Although intensity was generally described, frequency, total intervention duration, progression, supervision, and adherence were less consistently documented. Such variability limits the comparability of interventions and makes it difficult to determine whether particular exercise characteristics are more frequently associated with specific outcomes. Previous literature has highlighted the importance of systematically reporting exercise dose and prescription when evaluating exercise interventions in clinical populations [30,31,32,33].

The predominance of acute exercise studies is another relevant feature of the evidence map. Acute exercise may provide useful information regarding immediate changes in craving, affect, anxiety, or neurophysiological responses, but such findings cannot be directly extrapolated to longer-term adaptations associated with repeated exercise training. Longitudinal intervention studies with clearly defined progression and follow-up are therefore needed to complement the acute exercise literature [34,35,36].

### 4.2. Neurobiological and Biochemical Outcomes

Neurobiological and biochemical outcomes were investigated in a limited subset of the included studies, with particular attention to mechanisms related to reward processing and dopaminergic function. Measures included dopamine-related responses and dopamine D2/D3 receptor availability, among other neurophysiological variables.

The interest in dopaminergic mechanisms is consistent with the established role of mesocorticolimbic reward pathways in substance-related addictions. Chronic exposure to psychoactive substances has been associated with alterations in dopamine signalling and reward processing, which may contribute to craving, compulsive substance use, and relapse vulnerability [37]. Exercise has been proposed as a potential modulator of some of these pathways, and experimental and clinical literature has suggested that exercise-related neuroplastic adaptations may be relevant to reward processing and neurobehavioral recovery [38,39].

However, the evidence mapped in the present review remains insufficient to determine whether specific exercise prescriptions reliably modify neurobiological pathways in individuals with substance-related addictions. The studies differed considerably in exercise exposure, sample characteristics, neurobiological measures, and assessment timing. Moreover, some mechanistic evidence available in the broader literature derives from preclinical models, which were excluded from the present review because the current scoping review specifically focused on human intervention studies. Consequently, mechanistic interpretations should be regarded as hypotheses supported to varying degrees by the existing literature rather than as established effects of exercise in addiction populations.

### 4.3. Psychological and Addiction-Related Outcomes

Psychological and addiction-related outcomes represented an important domain of the mapped evidence. The included studies assessed variables such as anxiety, depressive symptoms, mood, affective responses, self-esteem, and craving. Some acute exercise interventions reported favourable changes in affective states or craving-related outcomes following exercise sessions [40,41,42].

These observations are broadly consistent with previous literature suggesting that exercise may influence affect regulation, stress responses, and psychological well-being through multiple interacting physiological and psychological mechanisms [43,44]. Nevertheless, the heterogeneity of the interventions and outcome measures prevents the identification of a consistent pattern across studies. In particular, findings obtained immediately after an exercise session should not be interpreted as evidence of sustained improvements in addiction-related outcomes.

The available evidence also suggests that exercise may be relevant to several dimensions of addiction treatment beyond the direct measurement of craving. Changes in mood, anxiety, affect, and self-esteem may be clinically relevant because these factors can influence treatment engagement and overall psychosocial functioning. However, the present evidence map does not allow conclusions regarding whether such changes translate into sustained reductions in substance use, relapse, or long-term treatment outcomes.

### 4.4. Heterogeneity and Methodological Considerations

One of the clearest findings of this scoping review was the considerable heterogeneity of the available evidence. The five included studies differed in study design, substance use disorder profile, participant characteristics, clinical setting, exercise modality, exercise intensity, intervention duration, and outcome assessment. This heterogeneity is not merely a methodological inconvenience; it reflects the early and developing nature of research on structured exercise interventions in addiction populations.

Sample sizes and study populations also varied considerably, and several studies evaluated acute exercise responses rather than longer-term training programmes. The representation of populations was uneven, with most studies focusing on adults with substance-related disorders in clinical or treatment-related settings. The limited number of studies and variation in outcome measures further restrict the possibility of identifying consistent associations between particular exercise prescriptions and specific neurobiological, psychological, or addiction-related outcomes.

Reporting of exercise prescription also requires improvement. The FITT components were not consistently reported with the same level of detail across studies, particularly with respect to frequency, progression, supervision, and adherence. Standardized reporting of these variables would improve reproducibility and allow future evidence syntheses to compare exercise dose and intervention characteristics more meaningfully.

The absence of a prospectively registered review protocol should also be acknowledged as a methodological limitation. Although protocol registration is not mandatory for scoping reviews, the lack of a registered protocol reduces the availability of an independent a priori record of the planned methods. In addition, the restriction of the literature search to publications from 2015 onward may have resulted in the exclusion of earlier intervention studies. This temporal restriction was selected to capture more contemporary research, particularly in the context of evolving approaches to addiction and exercise-based interventions, but it may have reduced the historical breadth of the evidence map.

### 4.5. Evidence Gaps and Future Research

The evidence map identifies several priorities for future research. First, there is a need for larger and better-characterized intervention studies using standardized reporting of exercise prescription. Future studies should systematically report frequency, intensity, session duration, exercise type, intervention duration, progression, supervision, and adherence.

Second, the current evidence base is concentrated on substance-related addictions. Although behavioural addictions were included within the scope of the review, no eligible human intervention studies addressing behavioural addictions were identified. This represents an important evidence gap and indicates a need for controlled exercise intervention studies in populations with behavioural addictions.

Third, the predominance of acute interventions highlights the need for adequately powered longitudinal randomized controlled trials examining sustained exercise training. Such studies should incorporate appropriate follow-up periods and evaluate whether changes observed during or immediately after exercise are maintained over time.

Fourth, future research should improve the standardization of outcome assessment. Studies combining validated addiction-related outcomes with psychological, physiological, and biochemical measures may provide a more comprehensive understanding of the potential role of exercise in addiction treatment. Where feasible, longitudinal studies should also assess clinically meaningful outcomes such as treatment engagement, substance use trajectories, craving over time, and relapse-related outcomes.

Finally, greater diversity in participant recruitment is warranted. Future research should include more balanced representation of women and men and consider different age groups, addiction profiles, treatment settings, and psychiatric comorbidities. Comparative studies of aerobic, resistance, HIIT, mind–body, and multicomponent exercise may also help determine whether specific exercise characteristics warrant further investigation.

### 4.6. Implications of the Evidence Map

Taken together, the findings indicate that exercise interventions have been investigated across several psychological, addiction-related, and neurobiological domains in individuals with substance-related addictions. However, the small number of eligible studies and their substantial heterogeneity prevent conclusions regarding the effectiveness of specific exercise modalities, intensities, or training doses.

The present findings should therefore be interpreted primarily as an evidence map. Rather than supporting a definitive therapeutic effect, the literature identifies potentially relevant domains—including craving, affective responses, psychological well-being, and neurobiological processes—that warrant further investigation using rigorous and adequately reported intervention designs. This distinction is particularly important when interpreting scoping review findings, whose primary purpose is to characterize the extent, nature, and gaps of available evidence rather than to establish treatment efficacy.

## 5. Conclusions

This scoping review mapped the available evidence on exercise training interventions in individuals with addictive behaviours, with particular emphasis on exercise prescription according to the FITT principles and neurobiological, psychological, and addiction-related outcomes. Following application of the eligibility criteria, only five human intervention studies were identified, all addressing substance-related addictions. No eligible intervention studies involving behavioural addictions were identified.

The mapped evidence encompassed different exercise modalities and intervention formats, including aerobic, combined, circuit-based, sport-based, and other structured exercise approaches. However, substantial heterogeneity was observed in exercise prescription, intervention duration, supervision, adherence, participant characteristics, and outcome assessment. Neurobiological, psychological, and addiction-related outcomes were investigated across different studies, but the limited number of studies and methodological heterogeneity prevent conclusions regarding the effectiveness of specific exercise modalities or FITT characteristics.

Overall, the current evidence base remains preliminary and fragmented. The findings therefore support the need for well-designed, adequately powered longitudinal intervention studies using standardized reporting of exercise prescription and clinically relevant outcomes. Further research is particularly warranted in behavioural addictions, which were included within the scope of this review but were not represented among the eligible intervention study.

## Figures and Tables

**Table 1:** Characteristics of the included studies and exercise interventions according to the FITT principles.

Study	Country	Design	Population/Addiction	Sample	Exercise Type	Frequency	Intensity	Time	Duration	Supervision	Adherence
Malagodi et al., 2024 [25]	Brazil	Acute crossover intervention	Hospitalized adults with SUD	43	Moderate-intensity aerobic and functional exercise	Single session	Moderate	~40 min	Acute	Supervised	NR
Ellingsen et al., 2023 [26]	Norway	Multicentre crossover intervention	Inpatients with poly-substance dependence	38	Soccer-based and circuit exercise	Single session	Moderate-to-vigorous	45 min	Acute	Supervised	NR
Hallgren et al., 2021 [27]	Sweden	Exploratory acute exercise study	Non-treatment-seeking adults with AUD	140	Cycle ergometer exercise	Single session	Strenuous; RPE ≈16	12 min	Acute	Supervised	NR
Welford et al., 2022 [28]	Sweden	Randomized controlled trial; secondary outcomes	Adults with AUD	140	Aerobic exercise and yoga	≥3 sessions/week	Moderate	60 min/session	12 weeks	Supervised	Monitored; exact adherence NR
Robertson et al., 2016 [29]	USA	Randomized controlled trial	Adults with methamphetamine use disorder undergoing behavioural treatment	19	Aerobic plus resistance exercise	3 sessions/week	Individualized moderate intensity	60 min/session	8 weeks	Supervised	NR

Legend: AUD, alcohol use disorder; SUD, substance use disorder; RPE, Rating of Perceived Exertion; NR, not reported.

**Table 2:** Outcomes assessed and main findings of the included intervention studies.

Study	Neurobiological/Biochemical Outcomes	Psychological Outcomes	Addiction-Related Outcomes	Principal Findings
Malagodi et al., 2024 [25]	Not assessed	Affect; mood state	—	Acute moderate-intensity exercise was associated with changes in affective response and mood state in individuals with SUD.
Ellingsen et al., 2023 [26]	—	Affect; anxiety; self-esteem	—	Acute exercise was associated with favourable changes in affective and psychological responses among poly-substance-dependent inpatients.
Hallgren et al., 2021 [27]	—	Mood; anxiety	Alcohol craving	Acute exercise was associated with reduced alcohol craving and favourable changes in mood and anxiety.
Welford et al., 2022 [28]	—	Depressive symptoms; anxiety	AUD-related clinical context	Exercise interventions were associated with changes in depression and anxiety outcomes in adults with AUD.
Robertson et al., 2016 [29]	Striatal dopamine D2/D3 receptor availability	—	Methamphetamine use disorder / behavioural treatment	Exercise training was associated with changes in striatal dopamine D2/D3 receptor availability during behavioural treatment.

## Data Availability

No new data were created or analyzed in this study. Data sharing is not applicable to this article.

## Abbreviations

The following abbreviations are used in this manuscript:

AUD	Alcohol Use Disorder
BDNF	Brain-Derived Neurotrophic Factor
D2/D3	Dopamine Receptor Subtype 2/Subtype 3
DA	Dopamine
FITT	Frequency, Intensity, Time, and Type
fNIRS	Functional Near-Infrared Spectroscopy
HADS	Hospital Anxiety and Depression Scale
HIIT	High-Intensity Interval Training
HRmax	Maximum Heart Rate
JBI	Joanna Briggs Institute
MeSH	Medical Subject Headings
min	Minutes
PANAS	Positive and Negative Affect Schedule
PCC	Population, Concept, and Context
PET	Positron Emission Tomography
PRISMA-ScR	Preferred Reporting Items for Systematic Reviews and Meta-Analyses Extension for Scoping Reviews
RCT	Randomized Controlled Trial
RPE	Rating of Perceived Exertion
STAI-Y1	State-Trait Anxiety Inventory—Form Y1
SUD	Substance Use Disorder
TH	Tyrosine Hydroxylase
VO_2_max	Maximal Oxygen Uptake
